# Going the Extra Mile: Why Clinical Research in Cystic Fibrosis Must Include Children

**DOI:** 10.3390/children9071080

**Published:** 2022-07-20

**Authors:** Rebecca Dobra, Siân Bentley, Claire Edmondson, Maxine Ovens, Clare Saunders, Christopher Short, Gemma Wilson, Jane C. Davies, Andrew Bush

**Affiliations:** 1 National Heart and Lung Institute, Imperial College, London SW3 6LY, UK; s.bentley@rbht.nhs.uk (S.B.); c.edmondson@imperial.ac.uk (C.E.); c.saunders@imperial.ac.uk (C.S.); christopher.short@imperial.ac.uk (C.S.); j.c.davies@imperial.ac.uk (J.C.D.); 2 Paediatric Respiratory Medicine Department, Royal Brompton Hospital, London SW3 6NP, UK; gemma.wilson15@nhs.net (G.W.); a.bush@imperial.ac.uk (A.B.); 3 Pharmacy Department, Royal Brompton Hospital, London SW3 6NP, UK; 4 Play Team, Royal Brompton Hospital, London SW3 6NP, UK; m.ovens@rbht.nhs.uk; 5 Centre for Paediatrics and Child Health, Imperial College, London SW7 2AZ, UK

**Keywords:** cystic fibrosis, children, clinical trials, ethics, patient-centred trials

## Abstract

This is an exciting time for research and novel drug development in cystic fibrosis. However, rarely has the adage, “Children are not just little adults” been more relevant. This article is divided into two main sections. In the first, we explore why it is important to involve children in research. We discuss the potential benefits of understanding a disease and its treatment in children, and we highlight that children have the same legal and ethical right to evidence-based therapy as adults. Additionally, we discuss why extrapolation from adults may be inappropriate, for example, medication pharmacokinetics may be different in children, and there may be unpredictable adverse effects. In the second part, we discuss how to involve children and their families in research. We outline the importance and the complexities of selecting appropriate outcome measures, and we discuss the role co-design may have in improving the involvement of children. We highlight the importance of appropriate staffing and resourcing, and we outline some of the common challenges and possible solutions, including practical tips on obtaining consent/assent in children and adolescents. We conclude that it is unethical to simply rely on extrapolation from adult studies because research in young children is challenging and that research should be seen as a normal part of the paediatric therapeutic journey.

## 1. Introduction to Cystic Fibrosis and the Evolution of Management

Cystic fibrosis (CF) is a multisystem disease inherited in an autosomal recessive fashion. People with two pathogenic variants in the cystic fibrosis transmembrane conductance regulator (CFTR) gene either fail to express CFTR protein or make CFTR with a defective function. Approximately 85% of those with CF have the F508del mutation on one or both alleles [1]. CFTR is an anion channel expressed on the surface of the epithelia lining the airways, gastrointestinal and reproductive tracts and sweat ducts [2]. Clinical manifestations result from salt (chloride, bicarbonate, sodium) and water transport abnormalities across epithelial surfaces [2,3,4] leading to: impaired mucociliary clearance and host defence defects in the airways and hence early bacterial infection and inflammation [5,6,7]; pancreatic exocrine insufficiency (PI) in 85% of patients, often present antenatally [5]; in utero failure of the vas deferens to develop leading to male infertility [5]; and multiple other systemic manifestations, including diabetes, liver disease and osteopenia [8,9]. The high concentration of CFTR channels lining sweat ducts makes measuring sweat chloride a feasible way of assessing CFTR function [10], and the diagnostic hallmark of CF is raised sweat chloride due to the failure of CFTR-mediated chloride reabsorption.

CF has been recognised as a clinical entity for decades [11], but since the discovery of the *CFTR* gene in 1989 and the understanding that subsequently evolved around disease pathogenesis, diagnosis, treatment and prognosis have changed dramatically [12,13,14]. Previously diagnosed following clinical presentation, many regions of the globe now have universal new-born screening (NBS) facilitating very early, often pre-symptomatic diagnosis of CF. The benefits of early diagnosis include improved nutritional outcomes, early initiation of treatments and surveillance for complications, and family awareness informing future reproductive decision-making [15,16].

All conventional treatments for CF target the downstream consequences of the disease and comprise: airway clearance with physiotherapy and mucoactive agents to aid expectoration; antibiotics; optimising nutrition with pancreatic enzyme supplementation; and surveillance for and treatment of specific complications such as CF-related diabetes. Over the last 10–15 years, there has been enormous progress made in a group of drugs that address the underlying defect in different classes of *CFTR* mutation [14]. Robust trials in people aged 12 years and over led to the licensing of 4 therapies, and data collection from trials is at various stages in younger children (Table 1). These therapies are “mutation specific”, i.e., they only work for certain mutation types. Mutation specific therapy has now been licensed for approximately 90% of the mutation combinations that cause CF. Clinical impacts include improvements in lung function, a decreased pulmonary exacerbation rate, an improved weight/BMI and quality of life [17,18,19,20].

Only one of these agents, ivacaftor, which targets approximately 5% of CF causing mutations, has been approved for infants, and it is currently licensed from 4 months of age. Therefore, priorities in CF research include establishing other agents’ safety and efficacy in young children to ensure these drugs are available as soon as possible after NBS diagnosis; this is particularly the case for the triple formulation, elexacaftor/tezacaftor/ivacaftor (ETI) given its substantial clinical efficacy and broad applicability [14]. Another unmet need is to improve symptom-directed therapies and establish optimal treatment regimens for: those ineligible for modulators; those who respond poorly; and those with established, irreversible disease at the commencement of therapy [14]. Novel approaches such as gene and mRNA therapy are entering early phase clinical trials in adults and although some way off, once safety and efficacy has been confirmed, focus will likely turn to younger populations with better preserved health [21].

## 2. *Why* Involve Children in CF Research

This is an exciting time for research in CF. When it comes to observational and interventional research, the adage, “Children are not just little adults” rings true. The following section explores *why* it is important to involve children in research.

### 2.1. Learning Things We Would Not Otherwise Know 

Involving children in research allows a better understanding of the early pathogenesis and progression of a disease. Synthesising data from studies in CF is complicated because of the different methods and treatment strategies. However, the key messages we have learned from studying young children are: lung disease starts early [22,23,24]; an absence of symptoms does not preclude significant detectable change in lung function [24]; early abnormalities persist into adulthood [25,26]; we are not treating early disease energetically enough [27]; and the clinical course can be improved by treatment [28,29]. Without paediatric research, we would not have been able to gain these important insights into CF disease progression and thus best management.

### 2.2. Preventing End Organ Damage

The key message for CF is that damage done in the pre-school years cannot be subsequently undone. This was demonstrated by a study comparing outcomes between the USA and the UK for patients homozygous for F508del. Spirometry at six years of age revealed that the USA patients had a 7% decrement in forced expiratory volume in 1 s (FEV_1_) relative to age matched controls but that this was 5% better than the Europeans [27]. Follow-up to age 18 years showed no catch-up of function in either cohort and an apparent further widening of the gap. In an observational study, only association, not causation, can be determined; however, the USA patients had been prescribed more rhDNase and hypertonic saline than their UK counterparts, suggesting that early aggressive treatment may be beneficial. This conclusion was supported by the Saline Hypertonic in Preschoolers (SHIP) study, which showed an improved lung clearance index in infants treated with hypertonic saline [28].

As CFTR modulator access increases, real-world evidence is demonstrating that the early initiation of treatment is interrupting disease progression in multiple systems. In the main studies involving children aged 5 years and younger, several participants had an increase in faecal elastase, sufficient to recategorise them from pancreatic insufficient to pancreatic sufficient [29,30], and in other studies children were able to stop pancreatic replacement therapy [31]. The evidence for the impact of early initiation of treatment on glucose tolerance is mixed [32,33,34,35], and the impact on the risk of ultimately developing CF-related diabetes is unknown [36].

Registry data in the UK has identified lower levels of infection with *Pseudomonas aeruginosa* and *Aspergillus* species in modulator-treated vs non-treated patients. Adolescents treated with long term lumacaftor/ivacaftor (over a 2-year period) showed improved lung function and nutritional status when compared to pre-treatment, although no improvement in pulmonary exacerbation rates or antibiotic usage were seen [37]. When compared with placebo, ivacaftor treatment of pre-pubertal children is associated with a significant increase in height velocity (>1 cm/year), suggesting additional benefits for linear growth in children who start treatment early [38].

In CFTR G551D knock in ferrets treated in utero with ivacaftor, there is a reduced incidence of meconium ileus, the male reproductive tract appears to be protected, and there is evidence of partial preservation of pancreatic exocrine function [39]. A recent case report identified a woman with CF who had taken ETI during pregnancy. Her baby was homozygous for F508del but had an immunoreactive trypsinogen (IRT) below the cut off on NBS, a normal faecal elastase and a relatively low sweat chloride (60 mmol/L) for this genotype. These findings suggested placental transfer of CFTR modulators with potential clinical impact and also ongoing post-natal exposure through breast milk [40]. Clearly significantly more work is required before any recommendations can be made based on these findings. However, if the work in ferrets and case reports does translate, this provides further evidence that early initiation of treatment may impact disease progression.

### 2.3. Extrapolating from Adults May Be Inappropriate or Misleading

It is widely recognised that medicines in children have differing pharmacokinetics (PK) to adults. These arise from the developmental changes that occur throughout childhood, which ultimately affect drug and metabolite levels, treatment response and the occurrence of adverse effects. Equally, the pharmacodynamics (PD) of a medicine—“what a drug does to the body”—requires careful study in children. There have been numerous examples over the years in which the consequences of the PD of a medicine in children could not have been predicted through adult studies, including growth suppression following the use of inhaled corticosteroids [41]. The relationship between the PK and the PD of a medicine is integral to ensuring that the dosing of medicines in children is safe and effective. However, despite these compelling factors, paediatric studies are still lacking for many drugs [42]. Without this information, dosing recommendations for children often are linearly extrapolated from adult dosing recommendations using weight or body surface area. Such an approach guesses at the age-related variability in the PK and the PD of a drug, with the potential for harm. In the absence of paediatric studies to support the licensing of medicines, the subsequent unlicensed use of adult formulations for children brings its own complications. There is a risk of error when manipulating adult formulations to administer smaller doses to children; as an example, crushing tablets to administer in a liquid form to a child may change the drug absorption and the PK, or it may render the drug unpalatable, adversely affecting adherence.

There is an important ethical question when it comes to novel drug licensing in children; do we just need to demonstrate safety or is it essential to prove efficacy as well? Some argue that if a drug is safe in children, and it has shown benefits in adults, then there is sufficient justification to prescribe it for children. However, others argue that before exposing a child to the potential side effects and inconvenience of a drug, we should be able to demonstrate that it will have clear benefits. This perhaps depends on the drug and its mechanism of action. Using CF as an example, dornase alfa acts by breaking down extracellular DNA in the airway lumen, reducing the viscosity and aiding clearance of mucus. The substrate for clinical efficacy, airway inflammation, will likely mean efficacy differences between adults (more established disease) and children/infants (earlier disease and healthier airways) [43]. In contrast, novel small molecule CFTR modulators improve the function of CFTR in all body systems affected in CF [44]. As the CFTR defect is present from the moment of conception, early restoration of function is considered beneficial, even in organs where it is difficult to measure, i.e., maintaining the healthy airway beyond infancy. It is likely an appreciation of this issue that led to licensing approval into early infancy of ivacaftor based on trials designed to confirm safety, PK and provide PD evidence of drug effect through sweat chloride measurements [29,30]. Measures of lung function were not required nor would they have had sufficient time or sensitivity to show efficacy in this age group. The finding of pancreatic exocrine benefits was initially unexpected but underscores the suitability of the approach being taken to study such drugs in this age group [30].

### 2.4. Rights and Fairness Issues

Involving children in research is an ethical minefield, particularly when it comes to interventional trials or more invasive observational trials. However, the 1989 “Convention on the Rights of the Child” recognises the right of the child to the “enjoyment of the highest attainable standard of health” [45]. Without age-appropriate studies to understand disease progression and to optimise treatment regimens in children, we cannot determine the best way to support children in the same right to evidence-based medicine as older children and adults. Development of medicines for use in children has been historically hindered by a lack of commercial interest from drug manufacturers due to the comparatively small market share children occupy [46]. This has improved over the last 20 years following the introduction of US and EU legislation [47] obligating and incentivising pharmaceutical companies to invest in paediatric research for both new and existing drugs. Major organisations across the globe have set out charters and guidelines to support and empower children and their families to talk about and take part in research. The Royal College of Paediatrics and Child Health (RCPCH) charter aims to support children, their families and healthcare professionals (HCPs) to discuss research in children [48].

## 3. *How* to Involve Children in Research

Now, we have hopefully justified the inclusion of children in research on several grounds, the second part of this article explores *how* to involve children and their families in safe and ethical research. It highlights some of the common challenges and presents potential solutions.

### 3.1. Choosing Suitable Outcome Measures

The utility of an outcome measure is determined by its feasibility, reliability and sensitivity for both an individual and a group. Therefore, as these properties change in different age groups and disease severity, the optimal outcome measure may show developmental differences. Young children may need different outcome measures to adults. Additionally, opportunistic data collection may be required where appropriate to minimise the frequency and the number of invasive procedures and to reduce the participation burden [49,50].

With the primary source of morbidity and mortality in CF due to lung disease, it is no surprise that lung function is often the primary outcome in clinical trials. The initial trials of modulators enrolled patients with established disease, FEV_1_ < 70%. Monitoring spirometry was thus an adequate endpoint. When trials were done in younger patients, or in those with normal spirometry, more sensitive tests such as multiple breath washout (MBW, below) had to be deployed. Assessing lung function or structure in the very early CF years (0–5 years) is challenging, as infants are unable to consciously perform a co-ordinated effort such as spirometry. Infants and, in some instances pre-schoolers, may need to be sedated for lung function testing or imaging which presents several ethical and practical considerations [51,52]. In children with minimal disease, it may take years to detect a signal showing that an intervention alters the outcome. Therefore, trials where the safety of an intervention is the primary focus (Phase I/II), feasible extrapulmonary outcomes are often utilised instead [53]. Mechanistic biomarkers such as sweat chloride and faecal elastase have been successfully used in CFTR modulator clinical trials [29,30,53]. Although a fall in sweat chloride is an excellent marker of a direct effect of modulator therapy on CFTR, it is not a marker of clinical efficacy and, on an individual basis, it correlates only poorly with measures of the latter. As adequate nutrition is the cornerstone of care in the early years, nutritional biomarkers can also be deployed such as z-scores for height, weight, and BMI [30]. Airway sampling for microbiology may also provide an endpoint of interest in trials [54]. In the adult population, historically, adequate sputum samples have been relatively straightforward for patients to produce, although this is becoming less simple since the advent of effective modulators. In children however, these samples can be hard to obtain as most pre-school children are unable to produce sputum on demand. Techniques such as sputum induction, or broncho-alveolar lavage can be used [22]. However, they are uncomfortable and, in the case of the latter, invasive, and can be difficult to justify ethically on a repeated basis. Suggested ways to minimise these procedures for children is to allow routine clinical investigations to be used as trial data, with flexibility from the trial protocol on when these samples need to performed and which laboratories they could be run in. Pragmatic registry trials such as CF STORM (https://www.cfstorm.org.uk/ (accessed on 14 June 2022)) have the other advantage of using clinical data from the registry, which avoids the cost and additional work of a trial database [49].

Despite its physiological limitations, spirometry, specifically FEV_1_ is still regarded as the gold standard lung function test. Spirometry requires a maximal co-ordinated effort, and although attempts can be made from 3 years of age it usually cannot be performed reliably until 6 years of age or older [55]. Even when children with CF can perform the manoeuvre reliably, it may still be within the normal range [56]. Furthermore, infants and pre-schoolers may expire their entire vital capacity in less than 1 s; FEV_1_ is therefore not an appropriate parameter in this age group. Alternative spirometric parameters are available for pre-schoolers and infants such as FEV_0.5_ or FEV_0.75_, but these have poor sensitivity and are not clinically validated [57,58].

**Figure 1:**
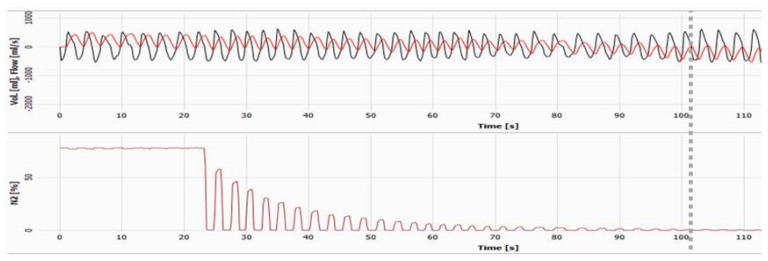
Multiple breath washout performed on a healthy subject. Top red line shows volume, whilst in black is the flow. The nitrogen (N_2_) trace shown in by the burgundy line, is used as the tracer gas, and it is “washed out” with 100% oxygen. During inspiration of 100% oxygen the N_2_ goes to 0%, whereas during expiration the breath is captured. N_2_ decreases in a step-like manner throughout the washout. Only 80 s of 100% oxygen breathing is required to reduce N_2_ down to the target concentration (dashed grey line = 2.5% of the starting N_2_ concentration hence LCI_2.5_). Subject has a normal LCI_2.5_ of 6.50.

An alternative lung function test which has gained substantial traction in recent years is MBW and its primary parameter, lung clearance index (LCI). MBW employs relaxed tidal breathing, where an inert gas is either washed into the lungs (to equilibrium) and washed out with room air or resident N_2_ is washed out with 100% oxygen. MBW measures ventilation efficiency and can be used successfully in non-sedated pre-schoolers but is highly dependent on the experience of the child and the operator(s) [59]. LCI is abnormal in young children with CF all the way through to adults, even when corresponding FEV_1_ values are within the normal range [60], and it has been used as the primary outcome in several recent clinical trials, showing positive treatment effects [61,62]. Figure 1 shows the MBW trace of a healthy child.

One weakness of MBW, its lack of structural information, can be complemented by imaging. The utility of CT in the early years has been shown most notably by the Australian Respiratory Early Surveillance Team for CF (AREST-CF) study. They reported early structural damage after NBS [63], although it should be noted that the London Cystic Fibrosis Consortium (LCFC) found CT to be a less reliable outcome measure [64]. Perth-Rotterdam Annotated Grid Morphometric Analysis for CF (PRAGMA-CF) aimed to develop a standardised outcome measure for assessing CF lung disease on CT of very young children with CF [65]. The regular use of CT is controversial due to the associated radiation burden [66,67]. In many contexts, needing to assess the response to an intervention predicted to act rapidly or for most clinical trials, frequent testing would be required for an imaging outcome to be useful. SHIP-CT is an example of using imaging in paediatric trials, and it demonstrated that the inhalation of 7% hypertonic saline had a positive effect on structural lung changes relative to isotonic saline. In contrast to CT, MRI is a radiation free imaging tool, and it is a promising clinical trial outcome in children [68]. However, the scanning time is longer than for CT and co-operation is required, meaning very young children may need general anaesthesia, which is clearly a drawback. Lung MRI is at an earlier stage in development, and it has not yet been fully standardised for multicentre research studies.

### 3.2. Environment and Team Skills

When implementing studies in children, it is essential to have sufficiently trained paediatric staff members. Study visits will often necessitate more than one member of staff to effectively manage more complex procedures. Children can be unpredictable and non-compliant, so staff need to be adaptable and flexible. The whole family are part of the clinic visit, not just the child. It is important to strike a balance between family involvement being advantageous rather than a hindrance.

A suitable environment is fundamental; a calm area, free from disturbance with enough space for the family to relax, will facilitate collection of the various measurements at a pace fitting to the child and testing schedule. Equipment can be noisy, cumbersome and intimidating, especially to the younger child, and it needs to be introduced by age-appropriate means. Certain procedures will understandably cause anxiety in both the child and the parent; extra time needs to be allocated to factor in the fretful child. A play specialist can be of value in such circumstances, for example by distracting the child during procedures such as blood sampling or facilitating age-appropriate preparation aids and sessions around any of the tests the child needs. To preserve the child’s willingness to participate in the measurements, tests with the potential for causing upset may need to be scheduled for the end of a visit.

### 3.3. Co-Design for Patient-Centred Research

Designing patient-centred research has been shown to: improve recruitment and retention; increase participant satisfaction; allow research teams to better meet participants’ psychosocial needs; prioritise outcomes that matter to patients; and enable a more diverse cohort to take part in trials [69,70,71,72]. This results in optimised time and cost-efficient research and reduced trial failure rates, and it ensures research cohorts are maximally representative of the actual clinical population.

There is sometimes a mismatch between what researchers think participants want from trial participation and what patients actually want [73]. Co-design of research recognises the expert opinion of patients and their families and helps to involve them in as many decisions as possible [74]. Many of the major research networks, including those specific to CF are setting up advisory groups, charters, and guidelines to assist with patient-centred research and co-design [75,76]. For example, the UK Cystic Fibrosis Trust’s Clinical Trials Accelerator Platform (CTAP) has a group of patient advisors who (i) provide insight into the feasibility of CF research to commercial and academic sponsors, and (ii) advise on ways to improve the research for patients. Invested stakeholders, i.e., children and their parents, should be involved in as many steps of designing the research, and at as early a stage as possible [48,69]. This may help to minimise assumptions on the part of researchers and identify any pitfalls in the project. An important part of co-design is ensuring all aspects of involvement are age appropriate. Children are likely to be less tolerant of unpleasant, time consuming, or poorly designed aspects of research participation [73,77,78]. Researchers should consider whether blood sampling is sufficiently minimised to ensure adequate cooperation from children and make sure that study materials, such as assent forms, use language accessible to the relevant age group [79]. Ensuring that research addresses the questions that are important to patients is also critical. A James Lind Alliance Priority Setting Exercise to identify the top 10 research priorities for people with CF was conducted in 2017 and the top priority was to reduce the burden of treatment regimens [80]. The exercise will be refreshed in 2022.

### 3.4. Approaching Oversubscribed Trials

Participation in trials with open-label extension phases and managed access programmes can allow children access to drugs before they are available to their age matched peers in clinic. Not all sites offering CF clinical care conduct trials. Inter-site referral and registry identification of eligible patients may reduce the problem, and CF research networks are working to streamline referral processes [81]. However, when a drug has been shown to be highly effective in adults, the number of families keen to enrol into the paediatric trial often far exceeds the number of available places. Allocation strategies for these competitive places include pragmatic approaches, e.g., selecting families who contact the team first, selection of patients with the greatest clinical need or random allocation. Pragmatic selection often results in a targeted selection of highly adherent families with a good knowledge of trials. These characteristics correlate strongly with factors such as socioeconomic status and health literacy, and this strategy will therefore compound inequity of opportunity [82,83]. Selection of patients with the greatest clinical need may result in an unrepresentative patient sample and produce data which is less relevant and/or generalisable. Random allocation may overcome this problem and result in a more diverse sample of patients entering trials, but many patients have a fear or dislike of the random element [84,85]. Our UK based study to identify what the CF community regard as the best or fairest way to allocate slots to competitive trials did not identify a universally acceptable or favoured solution [81]. Transparency and empathy surrounding the selection process may help to minimise any upset to families who are not selected.

### 3.5. Practical Challenges of Recruitment at the Time of Diagnosis

In the current CF landscape, there are two important trials that are recruiting patients at the time of, or shortly after, diagnosis. If the long-term goal is to optimise treatment regimens at diagnosis, when the child’s condition is optimal, studies recruiting at this time are imperative. The CF anti-staphylococcal antibiotic prophylaxis trial (CF START) is a randomised registry trial aiming to detect the safest and the most effective way of treating CF infants with antibiotics [86]. A second trial is the open label study assessing safety, pharmacokinetics and pharmacodynamics of long term ivacaftor treatment in CF infants (NCT03783286). To safely establish the correct dose and to match to subject weight, staged cohorts have been sequentially recruited, starting with subjects aged 12–24 months, subsequently 6–12 months and culminating in age 1–4 months. When recruiting so early after a diagnosis, the research team must approach the family with respect and understanding, when they are in the middle of processing life-altering news. Parents of older children may be more familiar with the many tests performed from clinic visits, but parents of new-born babies will be naïve to the numerous tests their child could undergo, a daunting prospect that requires careful explanation to allow families to choose the best option for them. It might be too much to contemplate at this time and they may decline the trial to reduce the burden on the child and the family. For some, however, trial participation can offer hope to a parent wanting their child to get access to treatment and allow parents to regain a sense of control.

*“There is an element of the unknown when deciding to allow your child into a clinical trial. However as hard as it is, medicines and procedures are sadly a very normal part of life with Cystic Fibrosis. We can never know how our child will react to any medicine (new or otherwise) but will have the peace of mind that the trials are heavily regulated and very well managed. Without the trials and the people who have taken part in them over the years, people with CF would not have access to the life changing drugs that are out there today. The existence of these trials gives my daughter the opportunity to live her life as happy and as healthy as she possibly can.”*

### 3.6. Impact of Participation on Schooling and Parental Work

Children with a chronic illness already miss significant amounts of schooling for hospital appointments and acute exacerbations compared to children without a chronic illness [87]. A UK based study to identify parent priorities and concerns revealed that parents are concerned about the impact of trial participation on children’s schooling. School is not only important for educational attainment but for the psychosocial well-being of children and adolescents [88].

Likewise, family members or care givers are required to attend trial appointments and investigations with the patient. This entails them having to take more time off work and to arrange childcare for other children. There are stipends in some trials for care-givers’ time but not usually for childcare. Trials can add a financial strain on the family, and possibly become a source of tension with employers [89]. Attempts to mitigate this include incorporating trial visits or investigations into children’s usual clinical appointments, using pragmatic trial designs, such as registry data collection or telemedicine, to minimise the number of visits to the site or increasing the stipend value. Flexibility in protocol windows and trial team availability may enable visits to be conducted after school or during holiday periods and to ensure that the same lessons are not regularly missed. Any extra inconvenience to the family and cost to the child by missing school is something that needs to be considered by ethics committees when reviewing a potential trial [90]. Communicating well with schools and employers may help them to understand why the trial is important and to provide appropriate support. Research teams should offer to liaise with schools and employers where desired.

### 3.7. Blood Tests and Invasive Procedures

In paediatric clinical practice, blood tests and invasive procedures are usually justified as being in the child’s bests interests for their medical treatment. In research, however, there cannot be a guarantee that participation will benefit the child, especially in trials where the patient may have been allocated to a placebo arm. Therefore, the risk-benefit balance can be impossible to determine. The dissent of a child should be respected in all aspects of research participation. Older children and adolescents can express their views on procedures, and if they refuse tests, this should be considered withdrawal of assent. However, most babies or toddlers will cry when undergoing procedures like blood tests, and it is very difficult to practicably determine whether this should be interpreted as withdrawal of assent.

In general, trials aim to minimise the number of invasive procedures with safeguards from ethics committees and Patient and Public Involvement in trial design [91]. Novel sampling methods, such as low volume assays, and opportunistic or pragmatic use of clinical investigations may help to minimise the number and the complexity of procedures.

### 3.8. Assent/Consent, Some Practical Tips

Although children can consent (or otherwise) for many treatments in their routine care, in clinical trials a parent or guardian must consent on the child’s behalf. The trial team must additionally gain the child’s assent and ensure they are given the opportunity to express their views on participation [89].

Assent and information forms should be tailored to the age of the child; however, these can sometimes still be too complex, especially for younger children. Some sponsors request that assent forms are signed by children as young as 3 or 4 years. At this age, many children are not able to read or to write their own names, and therefore this process seems futile, with much time spent trying to help a child sign a form that they are not able to read. Effort from the trial team is therefore better spent trying to verbally help the child to understand the trial. Emphasis should be less on the background to the research, and more on what will happen when they come to the trial visits, who they will be seeing, and what might happen to their bodies because of the intervention. Focus more on the what, less on the why. It is wise to get expert advice when planning how to communicate with very young children.

*“The consent forms are long. They’ve made the children’s one shorter and easier. The language is a bit easier to understand. But they asked my son to sign it as well as write his name. He’s 5. He doesn’t have a signature. He got really frustrated writing his name twice next to each other. I don’t think he’s the only one.”*

Older children are more likely to understand what is written in the assent forms, however, the forms can be long and boring for adolescents, who may not read them with the attention desired. When explaining the trial to an adolescent with CF, they will understand many of the different investigations as they will have experienced similar things with their usual CF care. Thus, the emphasis can be more on the why.

## 4. Conclusions

Although research in young children is challenging, it is unethical, and it may be misleading to simply rely on extrapolation from adult studies, and research should be seen as a normal part of the paediatric therapeutic journey. Medication PK may be different in children, and there may be unpredictable adverse effects. Children have the same right to evidence-based therapy as adults. Among the challenges of research in young children are measuring efficacy, especially when the disease is mild, and it may be only slowly progressive. It is essential to involve parents and, where age-appropriate, children in the planning of research to ensure that study visits are made as child-friendly as possible and trial investigations are acceptable. Paediatric trained trial staff and a child-friendly environment is essential. The CF community has been an exemplar of how to do research in children. First, large, definitive trials have been done in patients with established disease aged 12 and over. The next step has been trials in school age children and older patients with milder disease for which more sensitive endpoints, such as the lung clearance index, have been developed. Finally, trials are being done in preschool children and then babies. In this age group, efficacy trials would be prohibitively large and long to have adequate power, so surrogate endpoints, in the case of CF reduction in sweat chloride, have been accepted, together with PK and safety data, allowing the medications to be licensed. Furthermore, the unexpected benefits of early therapy, such as a reversal of pancreatic insufficiency, have been reported. Ongoing research in young children is essential if those diagnosed on NBS will be able to start evidence based, disease-modifying therapies at diagnosis, which should transform their long-term outlook.

## Figures and Tables

**Table 1:** Summary of licensed modulator characteristics.

Modulator	Mutation Profile	License Age in UK
Single agent ivacaftor	Gating mutations	≥4 months
Lumacaftor/ivacaftor	Homozygous F508del	≥2 years
Tezacaftor/ivacaftor	Homozygous F508del or F508del/residual function	≥6 years
Elexecaftor/tezacaftor/ivacaftor (ETI)	1 or 2 F508del mutations	≥6 years
